# Characterization and Adsorption Behavior of Newly Synthesized Aminated Cellulose with Jeffamine EDR148 Towards Ni(II), Cu(II), and Pb(II) Heavy Metal Ions

**DOI:** 10.3390/polym17020255

**Published:** 2025-01-20

**Authors:** Jawaher Y. Al Nawah, Amany S. El-Khouly

**Affiliations:** Department of Chemistry, College of Science, King Faisal University, P.O. Box 400, Al-Ahsa 31982, Saudi Arabia

**Keywords:** deoxychlorocellulose, aminated cellulose, jeffamine EDR148, metal ions adsorption, adsorption isotherm

## Abstract

Industrial wastewater containing heavy metal ions presents serious economic risk to the environment. In this study, a novel compound of aminated cellulose with jeffamine EDR148 was prepared to improve cellulose’s adsorptive behavior towards metal ions. This study undertook a straightforward and efficient cellulose modification through homogeneous chlorination in N,N′-butylmethylimidazolium chloride to produce 6-deoxychlorocellulose (Cell-Cl), followed by a reaction with jeffamine EDR148 and ultimately resulting in the formation of aminated cellulose (Cell-Jef148). Structural and chemical characteristics of Cell-Cl and Cell-Jef148 were determined using different techniques. Various adsorption conditions were applied to evaluate the optimal adsorption conditions for the removal of Cu(II), Ni(II), and Pb(II) ions. Cell-Jef48 revealed a greater affinity and higher adsorption efficiency of 480.3, 420.5, and 463.2 mg/g for Cu(II), Ni(II), and Pb(II) ions, respectively. Different kinetics and adsorption isothermal models were studied to investigate the adsorption mechanism and interactions between Cell-Jef148 and metal ions. The results fitted the Langmuir and pseudo-second-order models. Corresponding to the Langmuir model, Cell-Jef148’s maximum adsorption capacities were 952.38, 609.76, and 769.23 mg/g for Cu(II), Ni(II), and Pb(II) ions, respectively, with a high correlation coefficient, R^2^, in the range of 0.99575–0.99855. The research results of this study support Cell-Jef148’s adsorption of heavy metal ions, and the regeneration of adsorbent highlights the potential applications of cellulose-based materials in wastewater treatment.

## 1. Introduction

The rapid development in the worldwide industrial, petrochemical, and commercial markets has resulted in a rise of wastewater releases. This growth has resulted in the introduction of several contaminants into the environment that have an impact on living things [1,2]. This wastewater frequently carries a lot of pollutants, such as organic materials, dyes, and heavy metals that cause serious risks to human and animal health and valuable freshwater resources [3,4,5]. The presence of heavy metals can injure the kidneys, intestines, and liver, and possibly cause cancer [6,7,8]. As a result, various techniques, including membrane filtration [9], adsorption [10], natural processes [11], chemical precipitation [11], and electrochemical-based therapies [12], have been developed due to significant studies on the elimination of heavy metal ions. The adsorption techniques are becoming a better choice because of their low production costs, broad availability of raw materials, great commercial recycled percentage, and effectiveness in removing heavy metals [13,14].

Renewable resources and biomass materials have drawn more attention from academic researchers in recent years. Natural cellulose is the most prevalent polysaccharide found in nature, widely dispersed, and frequently used as a green material [15]. Cellulosic material is an inexpensive, renewable, and biodegradable raw substance. Cellulose as a linear homopolymer, consists of repeating units of β-D-anhydroglucopyranose connected by β-1,4-glycosidic linkages [16]. Chemically modified cellulose exhibits a greater capacity for adsorption than its untreated counterparts. The chemical modification of cellulose has shown success with several agents, including organic and inorganic acids, bases, and oxidizers [17,18,19,20,21]. However, it must provide novel adsorbents that are affordable, simple to make, and have a high adsorption efficiency in a range of examined parameters. The adsorption ability of functionalized cellulose with tetraethylenepentamine and chitosan towards Cu(II), Pb(II), and Cr(III) ions was examined. For Cu(II) ions, the Langmuir model yielded the highest optimal adsorption efficiency. The carboxymethylation technique was used to chemically modify textile fibers [22]. The removal of heavy metals from wastewater treatment using hydrothermally treated cotton, particularly cotton/polyester, has not been investigated with regard to Cd(II) and Pb(II) [23]. According to the results obtained using hydrothermal cotton/polyester, the equilibrium adsorption capacities for the adsorption of Pb(II) and Cd(II) ions varied from 2.06 to 19.98 mg/g and from 1.52 to 4.05 mg/g, respectively [23]. Cellulose isolated from sugarcane bagasse as an adsorbent has been employed in several studies, either in its raw or chemically treated form, where one of the studies showed that cellulose from sugarcane bagasse was able to remove Pb(II) and Ni(II) from wastewater with removal efficiencies of 89% and 91%, respectively [24]. The maximal adsorption capacity of polyethylenimine-modified sugarcane bagasse cellulose (SBCMP) was recorded to be 107.5 mg/g Cu(II) from wastewater. SBCMP was synthesized with a better removal efficiency of Cu(II), and the adsorption isothermal data fit the Langmuir model well [25]. To remove uranium (VI), polycationic (cellulose-NH_2_) and polyanionic (cellulose-COOH) cellulose beads were synthesized [26]. Cellulose-NH_2_ (at pH 6.0) and cellulose-COOH had the highest experimental adsorption capacities of 127.4 and 462.9 mg/g, respectively [26]. Cellulose was modified in the presence of the crosslinker N,N-methylenediacrylamide (MBA) and copolymerized with acrylic acid/acrylamide. The resultant P(AA-AM)@CNC adsorbent was capable of extracting 49.6 mg/g of Cu(II) from an aqueous solution [27]. A chitosan@diethylaminoethyl-cellulose (Cs@DEAECell) composite was investigated for its ability to remove Pb(II) and As(V) from water. The monolayer adsorption capacity of the composite was 41.98 mg/g for As(V) and 218.71 mg/g for Pb(II) [28]. Cellulose filament fibers were reacted with bisacrylamide by a cross-linking reaction to produce an amide-functionalized cellulose-based porous adsorbent. The obtained material showed a maximum adsorption capacity of 51.3 mg/g for Cu(II) [29]. Pendent methylbenzalaniline groups (DTD) on cellulose adsorbent were used to remove Pb(II) and Cu(II) ions. The modified cellulose has an adsorbent efficiency of 157.3 and 153.5 mg/g for Cu(II) and Pb(II), respectively [30]. Cu(II) and Hg(II) can be easily and quickly removed from aqueous solutions using cellulose treated with glutamic acid [31].

This study aims to explore the chemical modification of cellulose with jeffamine EDR148 and examine its adsorption properties as a bio-adsorbent for the elimination of Cu(II), Ni(II), and Pb(II) from aqueous media. As far as we know, no prior description or application of this kind of substance as a bio-adsorbent has been made. Cellulose cotton reacted with jeffamine EDR148 to produce a new, modified cellulose material. Cellulose was completely dissolved in BMIMCl ionic liquid and homogeneously reacted with thionyl chloride, resulting in a preparation of 6-chlorodeoxycellulose. Aminated cellulose with jefamine EDR148 was synthesized by the reaction of the obtained chlorodeoxycellulose with jeffamine EDR148. The adsorption efficiency of aminated cellulose with jeffamine EDR148 (Cell-Jef148) was examined under different adsorption conditions. This work could be helpful for future investigations into the cellulose-based synthesis of effective bio-sorbents.

## 2. Materials and Methods

### 2.1. Materials

Cellulose cotton and thionyl chloride (Wako Pure Chemical Industries, Osaka, Japan) were used without further purification. Jeffamine EDR148 (Jef148), ethyl acetate (Kishida Chemical Co., Osaka, Japan), N-methylimidazol, 1-chlorobutane, toluene (Kishida Chemical Co., Osaka, Japan), pyridine, sodium hydroxide, nickel chloride (NiCL_2_), copper chloride (CuCL_2_), and lead nitrate (PbNO_3_) were purchased from Wako Pure Chemical Industries, Ltd., Tokyo, Japan, and were used without further purification as well. To dissolve cellulose, an ionic solution of N,N-butylmethylimidazolium chloride (BMIMCl) was made as follows: 1-chlorobutane (1.38 mol) was combined with a stirred solution of 1-methylimidazole (1.25 mol) in toluene (125 mL, 1.356 mol) at 0 °C. After allowing the solution to reach room temperature, it was heated to 85 °C for 24 h under reflux and then frozen for another 24 h. To obtain a white crystalline solid, the semi-solid was decanted, cleaned with toluene, and then recrystallized from ethyl acetate. After drying, the resulting BMIMCl product had a yield of roughly 85%; proton (1H) NMR (400 MHz, chloroform-d) was used to characterize it.

### 2.2. Preparation of 6-Chlorodeoxycellulose

Ten grams of cellulose were dissolved in BMIMCl (100 gm) at 130 °C under vacuum conditions for 2 h. The solution was left at room temperature for one day, and the temperature was subsequently raised to 80 °C with stirring. To the clear homogenous solution, equimolecular concentrations of pyridine and thionyl chloride were added with stirring, and then the mixture was refluxed for 4 h. Thionyl chloride was added slowly at 80 °C while stirring and heating for 4 h. The product was washed with a diluted NaOH solution several times until pH 7 was reached; then, it was washed with distilled water several times. The yield was dried using a vacuum.

### 2.3. Amination of 6-Chlorodeoxycellulose with Jeffamine EDR148

In this study, 5.0 mL of jeffamine EDR148 reacted with 1.0 gm of chlorodeoxycellulose using mechanical stirrer under reflux conditions for 5 h at 30 °C. After some time, the solution was filtered, and the product was washed by dist. Water at pH 7. The sample was dried at room temperature (25 °C) in a vacuum for 24 h.

### 2.4. Characterization

With an accelerating voltage of 10 KV, the surface morphologies of modified cellulose samples were analyzed by SEM. The solid-state 13C-NMR investigation for the cellulose samples was performed. The FT-IR spectra of cellulose and its modified samples were measured to determine the functional groups. TGA was used to describe cellulose, Cell-Cl, and Cell-Jef148 in order to study their thermal stability. Also, the cellulose samples’ X-ray diffractograms (XRDs) were recorded to observe the change in the crystallinity of cellulose. Additional information regarding the conditions can be found in the Supplementary Materials.

### 2.5. Adsorption Experiments

The accurate weight of metal compounds was dissolved to provide a solution (50 mL) for the metal ions in dist. water. Moreover, 0.05 g of cellulose samples were added to the metal ion solution in each batch experiment. The metal ion concentration remained in the solution after a specified amount of time was ascertained using the UV–vis spectroscopy instrument (Hitachi U-3200 spectrophotometer, Hitachi High-Tech, Tokyo, Japan). Several factors that affect the modified cellulose samples’ adsorption effectiveness were examine: temperature (20–50 °C), time (0.5–36 h), medium pH (2.0–8.0) that was adjusted using 0.01 M HCl, and metal ion concentration (20–1200 mg/L). The adsorption capacity (q_e_) and the percent uptake (Pu) were determined using Equations (1) and (2) [32,33].

(1) $$q_{e}=\frac{C_{i}-C_{e}}{Wt}\;V\;\;\;\;\;\;\;\;\;\;\;\;\;\;\;\;\;\;\;\;\;\;\;\;\;\;\;\;\;\;\;\;\;\;\;\;\;\;\;\;\;\;$$

(2) $$Pu=\frac{C_{i}-C_{e}}{C_{i}}\times 100\;\;\;\;\;\;\;\;\;\;\;\;\;\;\;\;\;\;\;\;\;\;\;\;\;\;\;\;\;\;\;\;$$

where Wt = weight of the adsorbent sample (g), v = volume of the metal ion solution (L), and C_i_ = the initial concentration of metal ions (mg/L), and C_e_ = equilibrium concentration in the medium after certain time (mg/L). The whole adsorption tests were performed three times. The data collected were presented as mean ± SD.

### 2.6. Desorption and Reusability Process of Cell-Jef148

Adsorption/desorption processes were examined using 50 mg of Cell-Jef148 in 50 mL of metal ion solutions for 4 h at 25 °C. The desorption of Cell-Jef148 was eluted by 0.2 M of HNO_3_ solution, where Cell-Jef148 was added to the desorption solution for 4 h. This cycle was repeated 5 times with the same adsorbent. The remaining amount of metal ions in the medium was determined as discussed above.

## 3. Results and Discussion

### 3.1. Synthesis of Aminated Cellulose with Jeffamine EDR148

**Scheme 1:**
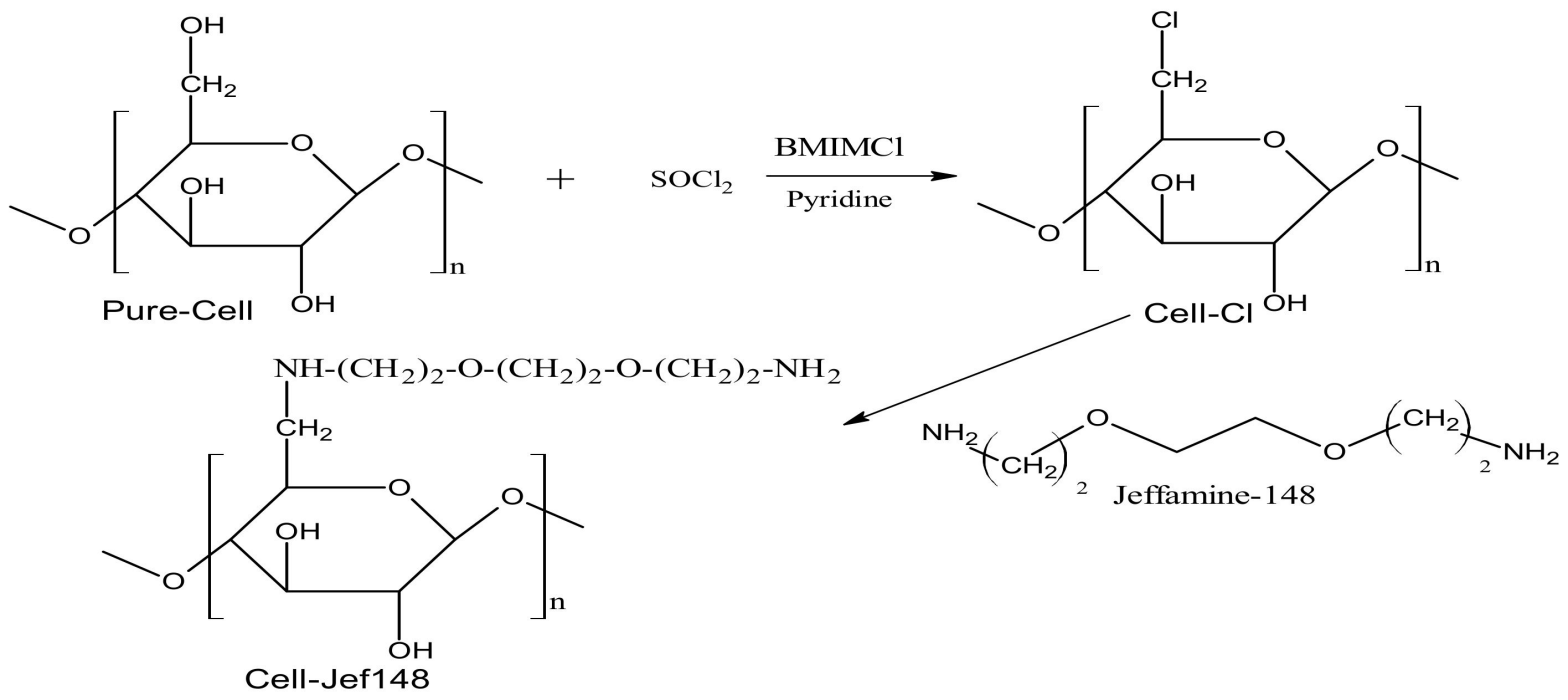
Preparation reactions of Cell-Cl and Cell-Jef148.

A new chemically modified cellulose adsorbent bearing jeffamine was synthesized in two steps, as shown in Scheme 1. Firstly, cellulose cotton (Pure-Cell) was dissolved in the BMIMCl ionic solvent and then reacted homogeneously with thionyl chloride (SOCl_2_) to form 6-chlorodeoxycellulose (Cell-Cl). Then, the obtained product (Cell-Cl) reacted with jeffamine EDR148 to produce aminated cellulose samples (Cell-Jef148). FT-IR, 13C CP-MAS NMR, SEM, X-ray, and TGA analyses were used to investigate the structural and chemical properties of Cell-Cl and its modified sample with jeffamine EDR148 (Cell-Jef148) in comparison to Pure-Cell.

### 3.2. Characterization

#### 3.2.1. SEM Analysis

**Figure 1:**
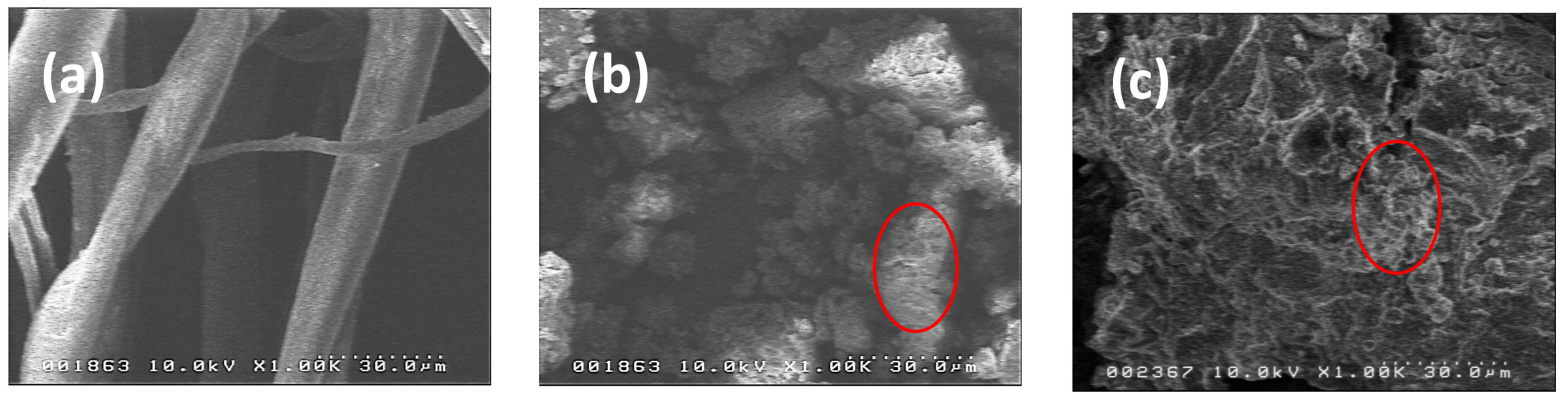
SEM images of (**a**) Pure-Cell, (**b**) Cell-Cl, and (**c**) Cell-Jef148.

The SEM technique was applied to examine the morphological and surface shape properties of Pure-Cell, Cell-Cl, and Cell-Jef148, as observed in Figure 1. Pure-Cell’s structure is distinguished by its smooth filaments and twisted fibers. After fully dissolving the cellulose sample in BMIMCl ionic liquid, the fiber structure was destroyed, and it reacted with thionyl chloride. Moreover, the Cell-Cl product was characterized by SEM analysis. The surface of Cell-Cl was reported to be uneven with aggregations, which are reasonably smooth bundles that resemble rocky surfaces. In contrast to Cell-Cl, Cell-Jef148 surfaces are more irregular and have rougher surfaces.

#### 3.2.2. FT-IR Spectra

**Figure 2:**
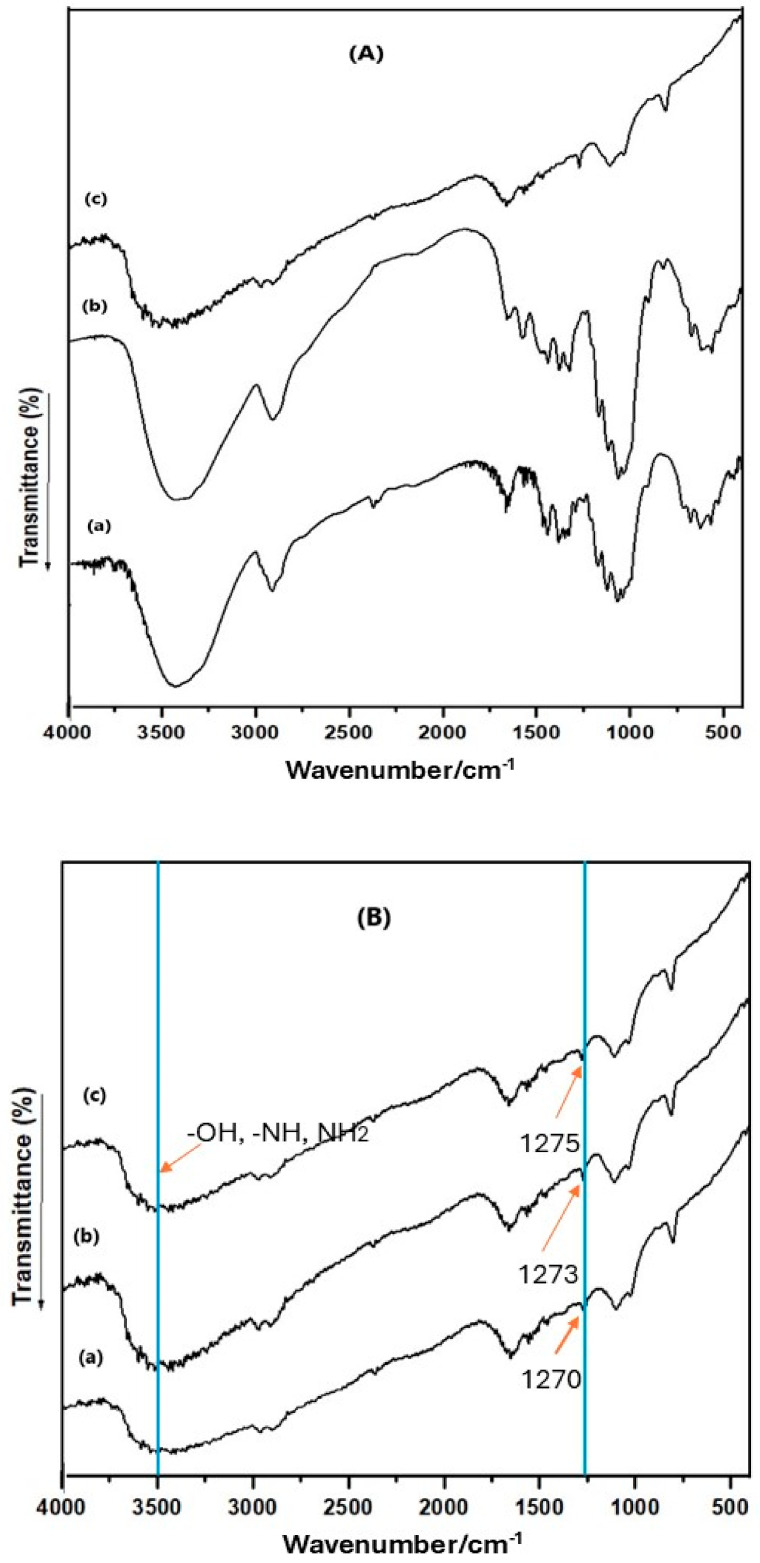
(**A**) FT-IR spectra of (a) Pure-Cell, (b) Cell-Cl, and (c) Cell-Jef148. (**B**). FT-IR spectra of Cell-Jef48 after adsorption of the metal ions, (a) Cu(II), (b) Ni(II), and (c) Pb(II).

FT-IR spectra of Pure-Cell, Cell-Cl, and Cell-Jef148 samples are presented in Figure 2. For Pure-Cell, a broad band at 3346 cm^−1^ is due to the stretching vibration of –OH groups (Figure 2A). Additional bands at 2988 and 1654 cm−1 might be attributed to vibration and stretching of C–H and C–C, respectively. The peak for cellulose’s C-O-skeletal vibration was found at 1058 cm^−1^. The FTIR spectrum of Cell-Cl showed an extra band at 750 cm^−1^ due to the C-Cl group. In the spectrum of Cell-Jef148, there are new bands recorded at 3450 cm^−1^ according to NH and NH_2_ groups in the jeffamine EDR148, which interact with the broad band of cellulose -OH groups. As a result, the broad band at 3340 cm^−1^ can be described as the stretching vibration of -OH, -NH, and NH_2_ groups. Another new band was observed at 1257 cm^−1^ due to the C-N group. The presence of all these bands in Cell-Jef148 confirmed the insertion of jeffmine EDR148 to the cellulose backbone structure.

The intensity of the peak related to NH and NH_2_ decreased after the adsorption of heavy metal ions, as shown in Figure 2B. Also, the peak at C-N group was shifted to 1270, 1273, and 1275 cm^−1^ after the adsorption of Cu(II), Ni(II), and Pb(II), respectively. These results indicate the interaction between Cell-Jef48 and heavy metal ions, revealing that amino groups have a strong effect on the adsorption of heavy metal ions on the surface of cellulose materials.

#### 3.2.3. ^13^C-MS-Mass-NMR Spectra

**Figure 3:**
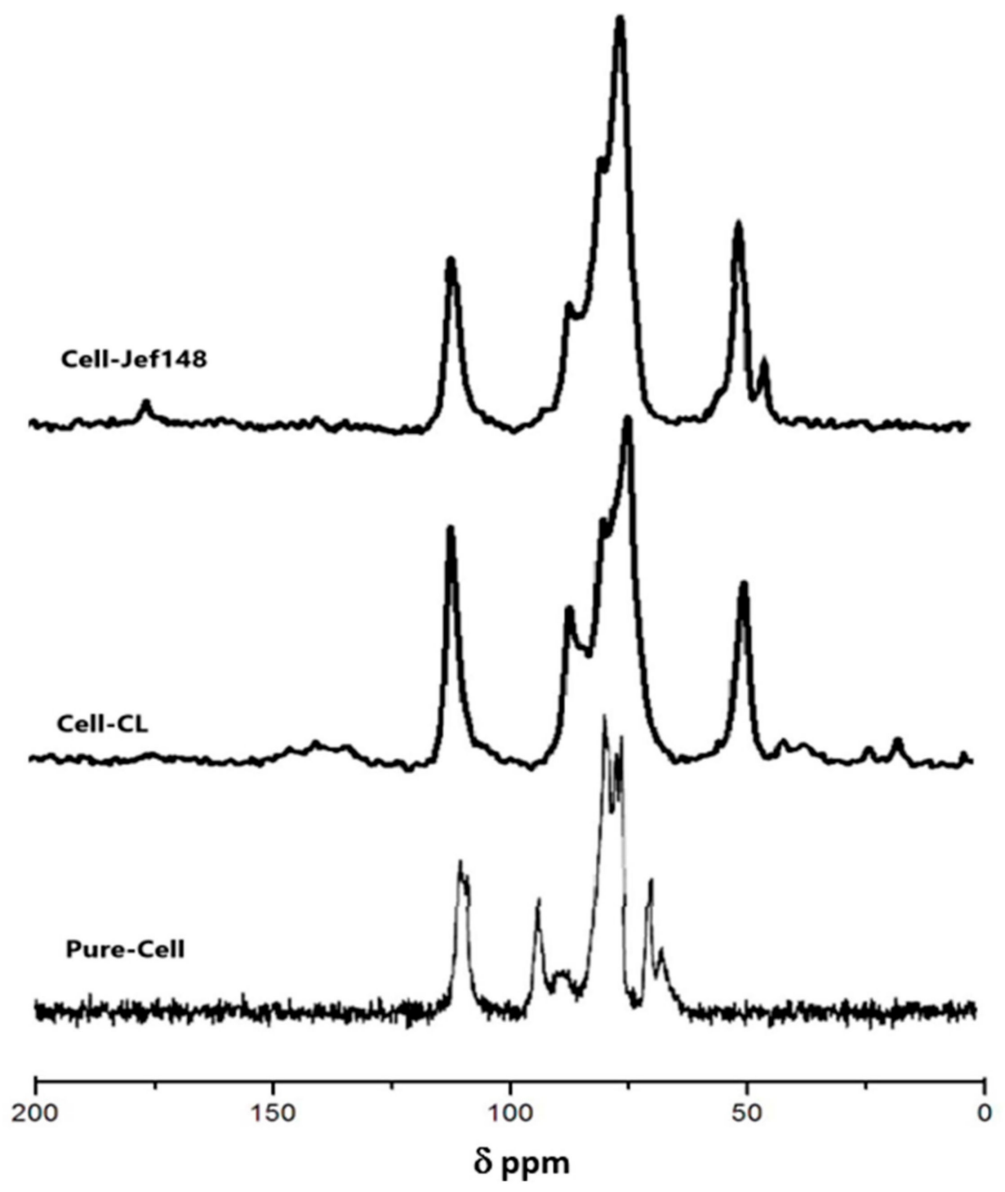
^13^C CP MAS NMR spectra of Pure-Cell, Cell-CL, and Cell-Jef148.

Figure 3 depicts the ^13^C-MS-Mass NMR spectra of Pure-Cell, Cell-Cl, and Cell-Jef148. The characteristic peaks of cellulose were observed in all samples. The spectrum of cellulose showed peaks of C-1 at 110 ppm; C-4 at 86 ppm; C-2, C-3, and C-5 at 74.5 ppm; and C-6 at 64.5 ppm [34]. The chemical shift of C-6 to 45.4 ppm was noticed in the Cell-Cl spectrum. The chemical shift of C-6 changed significantly from 64.5 to 45.4 ppm when the –OH group was partially replaced by chloride at C-6. While there was no significant effect on the chemical shift of C-2, C-3, or C-5, the replacement only slightly altered the chemical shift of C-1 from 110 to 111.5 ppm and C-4 from 86 to 81.3 ppm [35,36]. These results confirm the introduction of Cl onto cellulose structure. With the reaction of Cell-Cl with jeffamine EDR148, new peaks appeared indicating the reaction and the formation of the covalent bond of jeffamines with cellulose. In the ^13^C-NMR spectrum of Cell-Jef148, the corresponding carbons of O-CH_2_- and N-CH_2_ are found at 45.2 ppm and 72.3 ppm, respectively, which interact the carbon peaks of cellulose. All of the results confirm the successful reaction between cellulose and jeffamine EDR148.

#### 3.2.4. X-Ray Spectra

**Figure 4:**
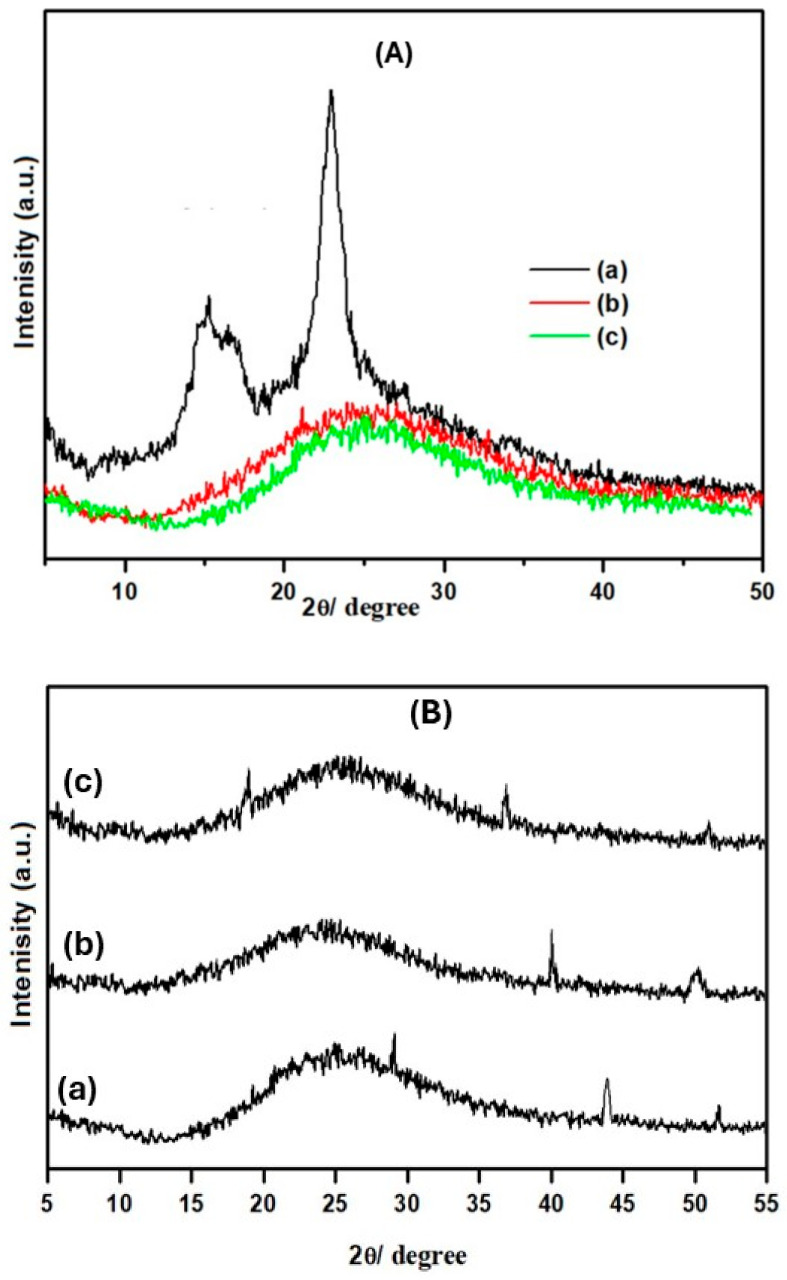
(**A**) X-ray diffraction of (a) Pure-Cell, (b) Cell-Cl, and (c) Cell-Jef148. (**B**) X-ray diffraction of Cell-Jef148 after the adsorption of metal ions (a) Cu(II), (b) Ni(II), and (c) Pb(II).

X-ray diffraction spectra of Pure-Cell, Cell-Cl, and Cell-Jef148 are shown in Figure 4A. Three characteristic peaks that correspond to the 101, 10l’, and 002 planes in Pure-Cell’s X-ray diffraction are visible [37,38]. On the contrary, Cell-Cl shows additional crystalline peaks, but with less intensity than those seen in pure cellulose. These peaks indicate that the residual hydroxyl groups and the bound chlorine atoms create a new composition [32,33]. When further modifications, such as attaching jeffamine EDR148 to the backbone, are implemented, the reduced crystallinity of Cell-Cl becomes particularly intriguing. Figure 4 illustrates the significant decrease in crystallinity for Cell-Jef148 in contrast with Pure-Cell and Cell-Cl. According to these results, modified cellulosic samples are shown in amorphous forms, and the attachment of new groups to the cellulose backbone does not form an ordered structure. Figure 4B shows X-ray diffraction of Cell-Jef148 after adsorption the metal ions. As observed, there are new peaks with slight intensity that appeared, which are related to Cu(II), Ni(II), and Pb(II) metal ions [39,40]. X-ray of Cell-Jef148-adsorbed Cu(II) metal ion shows new peaks of Cu(II) at 2θ of 28.2, 44.5, and a slight one at 52. In the X-ray spectrum of Cell-Jef148-adsorbed Ni(II), the new peaks at 2θ of 40.3 and 51.1 correspond to Ni(II). Also, the peaks at 2θ of 19.2 and 38.9 in the spectrum of Cell-Jef148-adsorbed Pb(II) are related to the Pb(II) metal ion. As a result, these peaks confirmed the adsorption of the metal ions onto the surface of Cell-Jef148 as a highly effective adsorbent.

#### 3.2.5. TG Analysis

**Figure 5:**
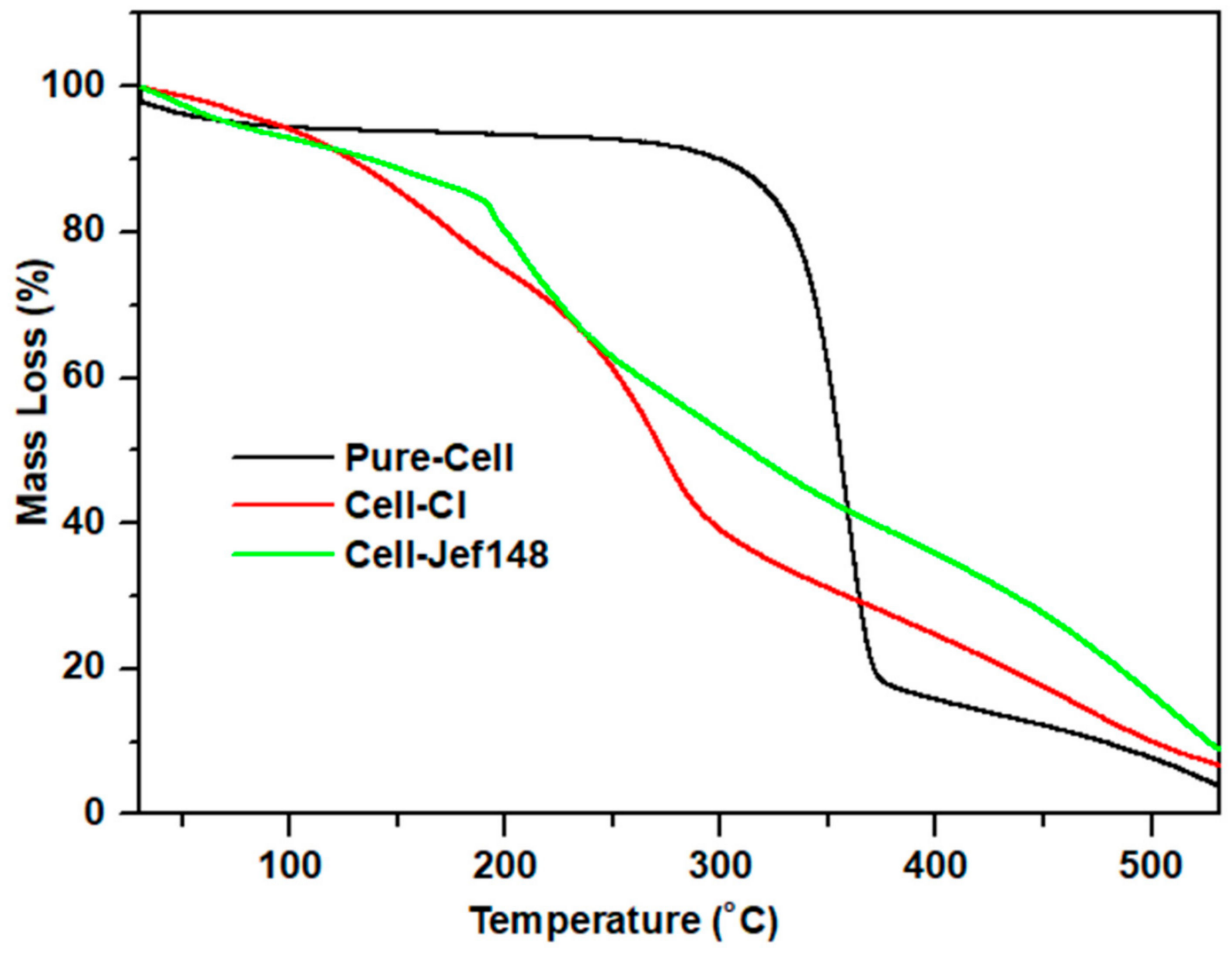
TG graphs of Pure-Cell, Cell-Cl, and Cell-Jef148.

In Figure 5, thermogravimetric analyses of Pure-Cell, Cell-Cl, and Cell-Jef148 were displayed. According to thermogravimetric curves, Cell-Cl and its modification, jeffamine EDR148, Cell-Jef148, showed less heat stability than pure cellulose. Cellulose remains stable up to 250 °C and begins to decompose between 250 and 380 °C with a 92% mass loss due to the pyrolytic decomposition of the carbon backbone. After the introduction of Cl atom to cellulose backbone, the degradation rate decreased in Cell-Cl to be in the range of 150–280 °C, compared to the degradation rate of pure cellulose with 80% mass lass. Additionally, Cell-Cl showed two degradation stages. The first stage is in the range of 150–250 °C, due to the C-Cl bond cleavage and condensation of OH groups on C-2 and C-3 of cellulose. The other decomposition stage, in the range of 250–350 °C, corresponds to the degradation of the cellulosic chain [36,40,41,42].

The TG graph of Cell-Jef148 recorded degradation stage in the range of 100–190 °C with a weight loss of 8.2%, indicating simple degradation of cellulose structure at C-2 and C-3. Another significant weight loss of 41% was noted in the 100–350 ◦C range. This can be attributed to the essential stages of cellulose’s thermal degradation or depolymerization, which occurs when glycosidic linkages (C–O–C) break down with the breakdown of the loaded aminated chain. Weight loss decreased slightly between 350 °C and 530 °C, with 3.5% residue left in Pure-Cell, 6.4% in Cell-Cl, and 9.5% in Cell-Jef148. This could be attributed to the complete degradation of the cellulose backbone. Furthermore, some authors have suggested that the insertion of a heteroatom as N may be responsible for a minor rise in the residue that could lead to formation by-product along with charred residue [43,44,45]. These findings indicate the disruption of the crystalline region of pure cellulose in Cell-Jef148, which is in agreement with the X-ray analysis discussed. Therefore, the disruption of the crystalline region, more amorphous, increases the surface activity towards the removal of the metal ions from the aqueous solution.

### 3.3. Adsorption Behavior of the Modified Cellulose Towards Heavy Metals

According to our previous work, pure cellulose has low adsorption affinity toward heavy metals [36]. Moreover, studying the adsorption efficiency of Cell-cl for the heavy metal ions showed low ability as in pure cellulose, as shown in Figures S1–S4 in Supplementary Materials. The maximum adsorption removal efficiency of pure cellulose was 5.2, 4, and 4.2% for Cu(II), Ni(II), and Pb(II), respectively. In the case of Jell-Cl, the maximum adsorption efficiency for removal was 12.5, 10.5, and 11.8% for Cu(II), Ni(II), and Pb(II), respectively. To improve the cellulose affinity, Cell-Cl was modified to be functionalized with jeffamine EDR148. Here, we plan to investigate how various experimental settings affect heavy metal adsorption, i.e., Cu(II), Ni(II), and Pb(II), on the surface of Cell-Jef148.

#### 3.3.1. Effect of pH Medium on the Adsorption

**Figure 6:**
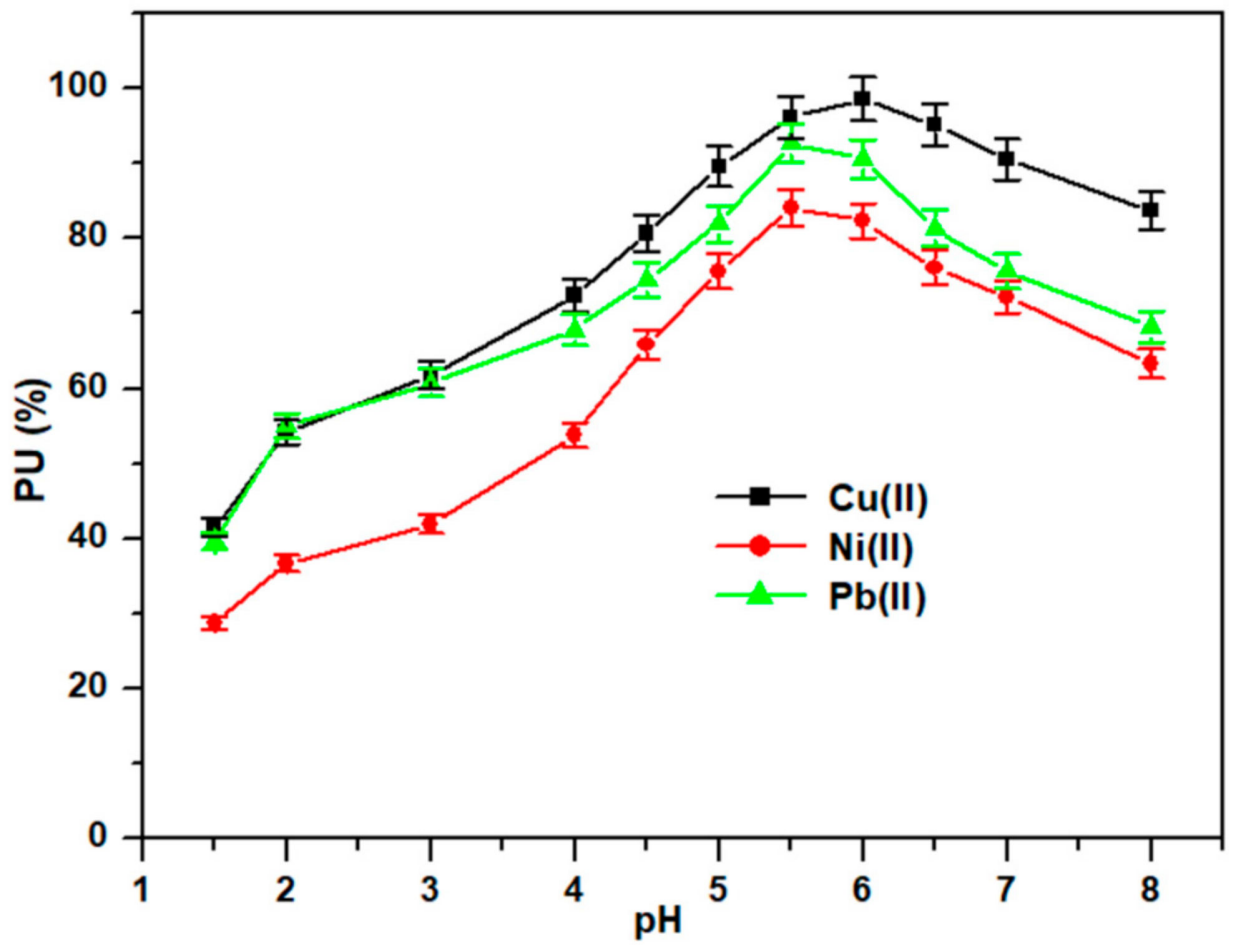
Influence of pH on the adsorption of heavy metal ions.

The pH medium affected the adsorption efficiency of Cell-Jef148 towards the heavy metal ions as shown in Figure 6. The effect of pH on the Cell-Jef148’s capacity to adsorb Cu(II), Ni(II), and Pb(II) ions of 500 mg/L metal ion concentration from an aqueous solution was investigated in the pH range of 1.5–8.0 at preliminary test conditions of temperature 25 °C, time of 4 h, 50 mL volume of adsorption medium, and adsorbent of 50 mg (Figure 6). The pH of the medium was adjusted before the addition of Cell-Jef48 adsorbent using 0.01 M hydrochloric acid. The maximum adsorption amounts of metal ion onto Cell-Jef148 were detected at pH 5.5 for Ni(II) and Pb(II) and at pH 6.0 for Cu(II). The adsorption capacities exhibited low levels at reduced pH values while demonstrating higher levels at increased pH values. According to the results, adsorption capabilities were high at higher pH levels and poor at lower pH values. Since the amine function is strongly linked to hydronium ions (H_3_O^+^) at lower pHs, the approach of metal cations is restricted due to the repulsion force, and as a result, the metal ions in solution cannot be bound. The adsorption surface becomes deprotonated when the pH rises, which increases the possibility of electrostatic attraction from amine groups providing electron pairs [46,47]. As a result, the complex reaction that these functional groups participate in during the metal ion absorption process causes the amount of adsorbed to rise. Moreover, the adsorption effectiveness began to decrease at increasing pH levels. The reduction in adsorption efficiency could be caused by the production of metal hydroxides and oxides at pH values greater than 6.0. Consequently, the optimum pH 5.5 value was applied to each of the following tests.

#### 3.3.2. Effect of Temperature and Contact Time on Adsorption Efficiency

**Figure 7:**
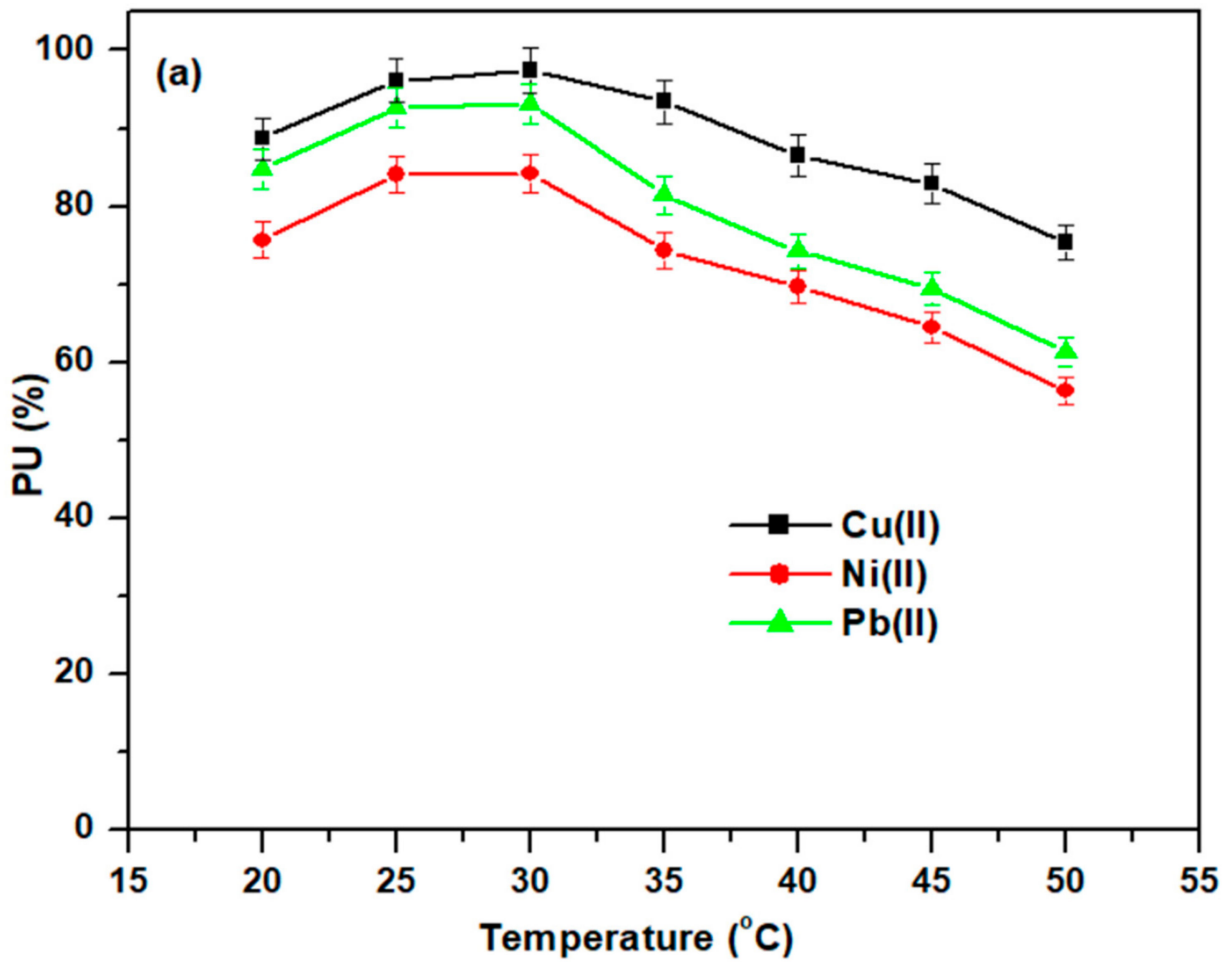

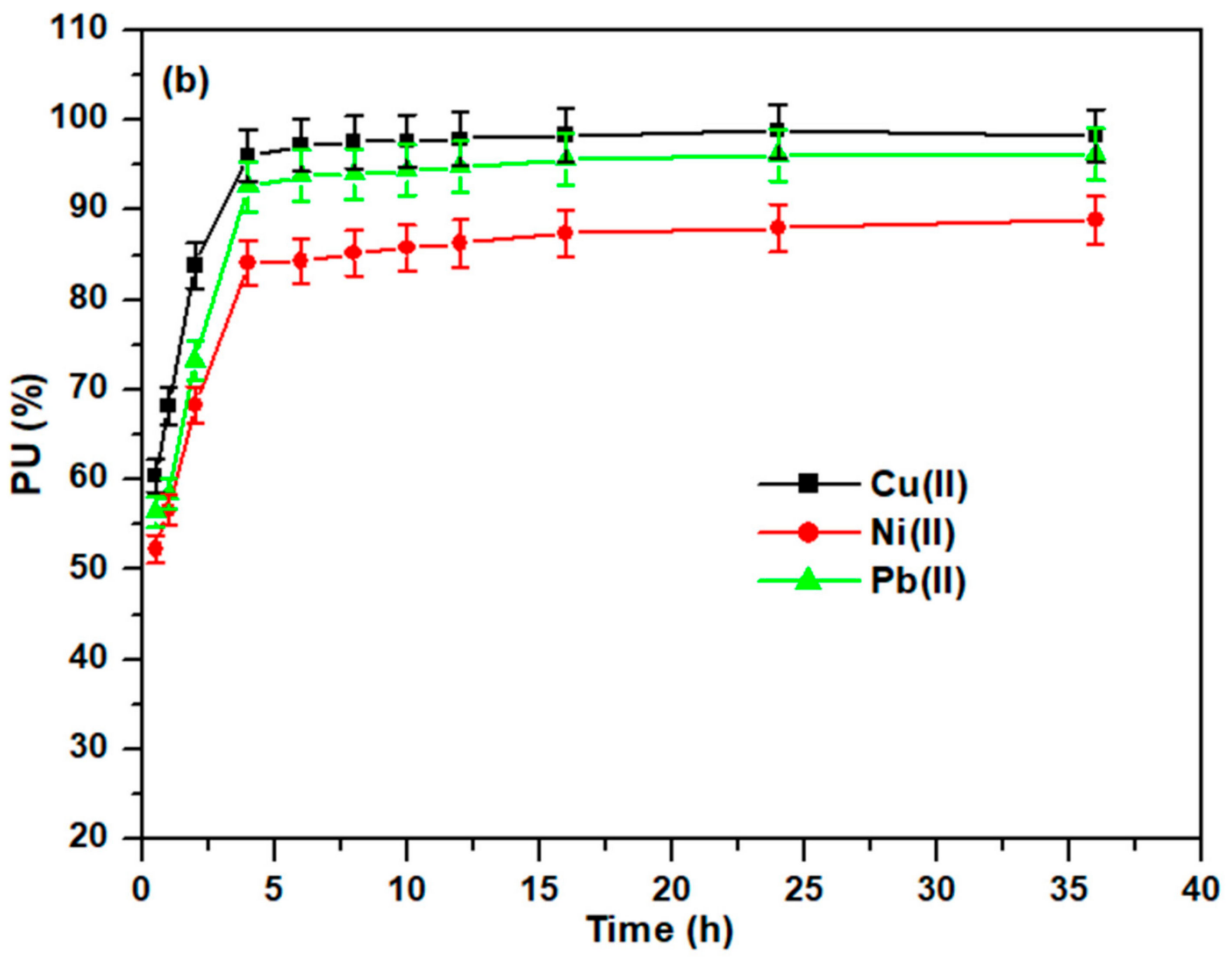
(**a**) Influence of temperature and (**b**) on the adsorption of heavy metal ions.

The adsorption efficiency of Cell-Jef148 towards the metal ions is significantly affected by the adsorption reaction temperature at adsorption parameters of 50 mg of the adsorbent, 500 mg/L metal ion concentration, 50 mL volume of the adsorption solution, time of 4 h, and pH 5.5. Figure 7a shows the adsorption capacities of Cell-Jef148 for Cu(II), Ni(II), and Pb(II) metal ions during the temperature range of 20–50 °C. It was observed that after 25–30 °C, on the surface of the modified cellulose sample, the metal ions’ adsorption decreased, suggesting that their diffusion rate was slower and their desorption rate was faster than their adsorption rate. For these heavy metal ions, lower temperatures—specifically, 25 °C—are ideal for generating the best adsorption efficiencies due to the exothermic behavior of the adsorption procedure.

As illustrated in Figure 7b, the removal of Cu(II), Ni(II), and Pb(II) metal ions by Cell-Jef148 at various durations was investigated under adsorption reaction conditions of 50 mg of the adsorbent, 500 mg/L metal ion concentration, 50 mL volume of the adsorption solution, temperature of 25 °C, and pH 5.5. The removal efficiency of the heavy metal ions increased until 4 h of adsorption time. At 4 h, the maximum removal efficiency values were 96.06%, 84.2%, and 92.64% for Cu(II), Ni(II), and Pb(II), respectively. The adsorption behavior changed quite quickly in the beginning and then gradually attained equilibrium adsorption in 4 h. Since the adsorption equilibrium was reached in 4 h, increasing the adsorption duration after 4 h has no effect on the removal efficiency, which slightly increases with no significant effect or remains unchanged. The huge number of empty active sites on the adsorbent was occupied quickly by metal ions within 4 h; hence the removal efficiency was not significantly increased after that time.

#### 3.3.3. Effect of Adsorbent Dose on the Adsorption Rate

**Figure 8:**
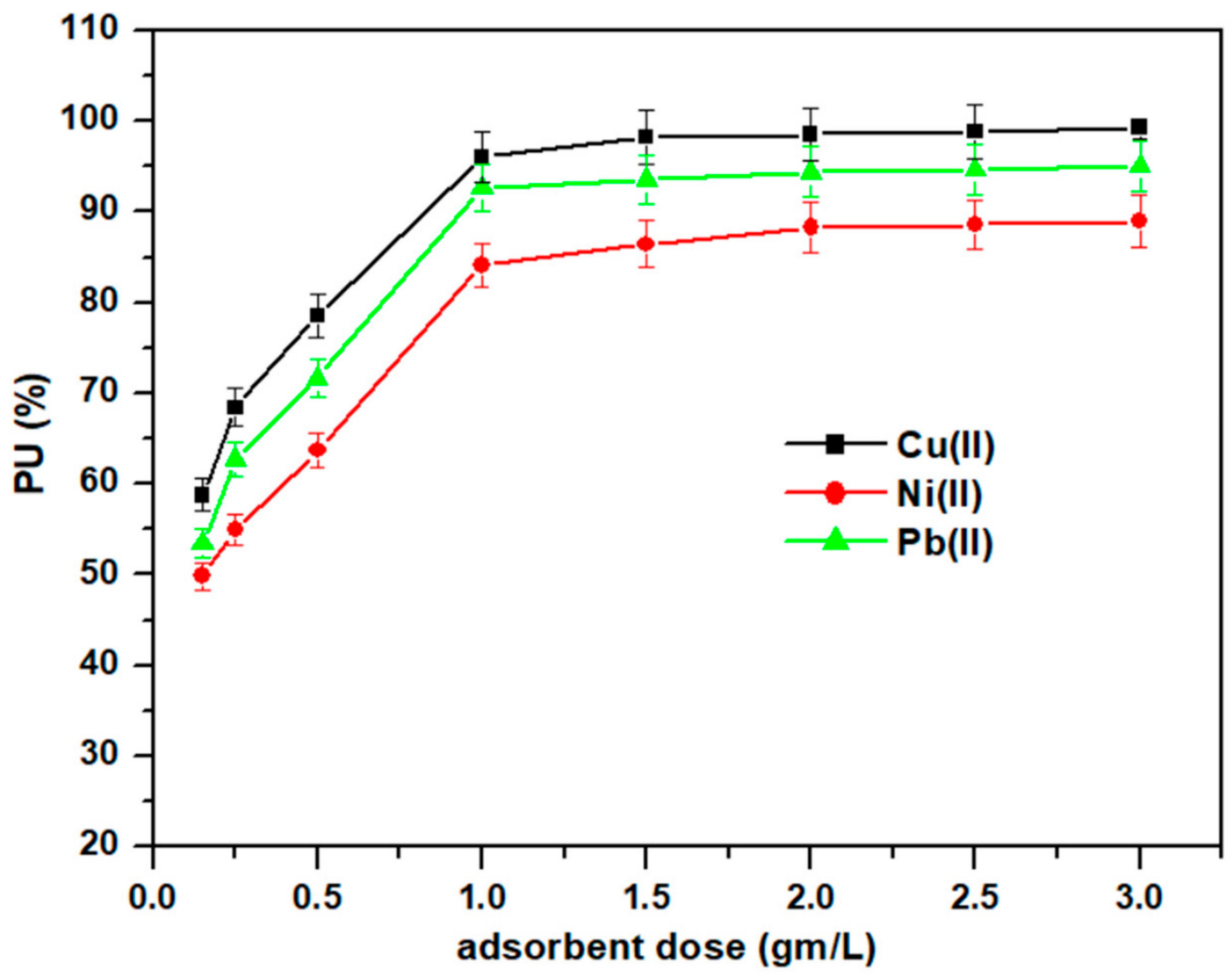
Influence of adsorbent dose on the adsorption of heavy metal ions.

The amount of adsorbent is considered one of the most effective factors for the adsorption efficiency towards the heavy metal ions at adsorption conditions of 500 mg/L metal ion concentration, 50 mL volume of adsorption solution, temperature of 25 °C, time of 4 h, and pH 5.5. Figure 8 shows the effect of Cell-Jef148 dosages with a range of 0.025–0.125 gm in 50 mL of experimental solution as 0.15–2.5 gm/L adsorbent. As observed, the amount uptake of Cu(II), Ni(II), and Pb(II) increased with an increase in Cell-Jef148 amount up to 1 gm adsorbent dose due to increasing the quantity of the active binding sites in the solution. The equilibrium adsorbent dose was 0.05 gm (1 gm/L) for metal ions Ni(II) and Pb(II). The percent uptake was 84.1% and 92.64%, respectively, and also the equilibrium adsorbent dose for Cu(II) was 0.05 gm (1 gm/L) with a percent uptake of 96.06%. The rare increase or constant value of the percent uptake after the equilibrium adsorbent dose can be a result of the active adsorptive sites on Cell-Jef148 not being fully occupied with the metal ions. Accordingly, the optimum Cell-Jef148 dose was around 0.05 gm (1 gm/L), which was tested in the remaining factors examined.

#### 3.3.4. Effect of Metal Ion Concentration on the Adsorption Rate

**Figure 9:**
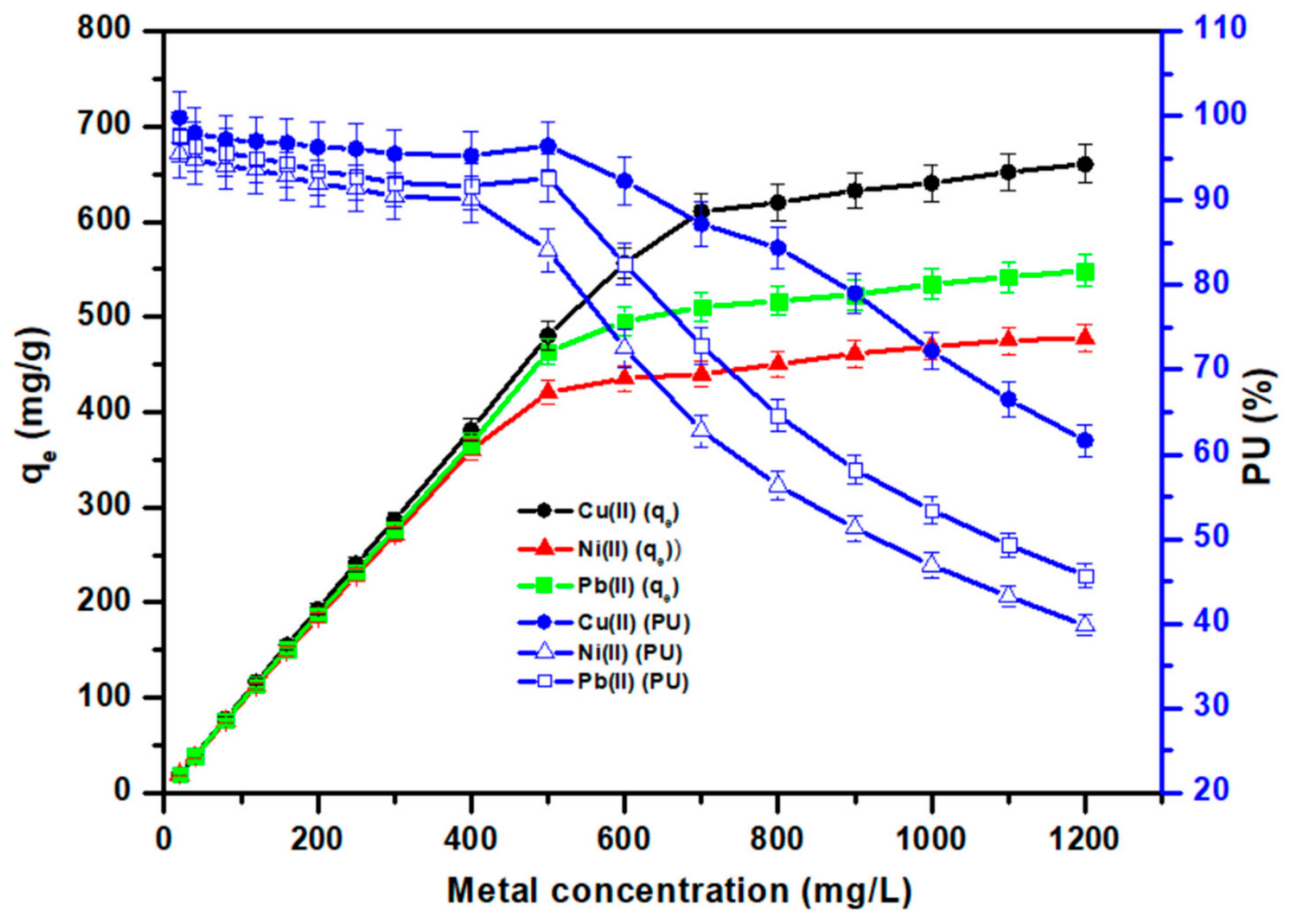
Influence of metal ion concentration on the adsorption of the heavy metal ions.

Figure 9 represents the adsorption capacity and percent uptake measured using Cell-Jef148, with metal ion concentrations of 20–1200 mg/L while maintaining constant the adsorbent’s dosage, duration, pH, and temperature as mentioned above. As shown in Figure 9, the results show that Cell-Jef48′s adsorption capacity increased significantly with higher initial metal ion concentrations, revealing a linear increase in manner at lower concentrations and an equilibrium at higher concentrations. With an increase in metal ion concentration up to 500 mg/L for Ni(II) and Pb(II) and 700 mg/L for Cu(II), Cell-Jef148 was able to absorb metal ions. The adsorbent’s active sites were satisfactory for holding the metal ions at low concentrations. The adsorption efficiency of Cell-Jef148 drops as the concentration of heavy metal ions is increased above the optimal values where the adsorbent Cell-Jef148 has no capacity to absorb extra molecules. The adsorption capacity remains almost constant after the optimal initial concentration of metal ions, and percent uptake decreases with rising metal ion concentration. This suggests that Cell-Jef148’s active sites reduce, which prevents the adsorbent from picking up more amounts of metal ions. For Ni(II) and Pb(II), Cell-Jef148’s adsorption capabilities were 420.4 mg/g (Pu of 84.1%) and 463.2 mg/g (Pu of 92.64%), respectively, at the initial metal concentration of 500 mg/L. Additionally, at the initial metal ion concentration of 500 mg/L, Cell-Jef148 had adsorption capacity of 480.3 mg/g (Pu of 96.02%) for Cu(II) ions.

#### 3.3.5. Adsorption Isotherm Studies

The adsorption technique (mechanism) of Cell-Jef148 was modeled and explained in this study using the Freundlich isothermal adsorption model, the Langmuir isothermal adsorption model, and the Temkin isothermal model, respectively, in order to investigate the adsorption behavior between the adsorbent and the adsorbate. At optimal adsorption conditions, the corresponding parameters of the Langmuir, Freundlich, and Temkin models were established for Cu(II), Ni(II), and Pb(II) ions with metal ion concentration ranges of 200–500 mg/L. The Freundlich isothermal adsorption model is typically applied to materials with inhomogeneous surfaces and shows how chemo-physical adsorption controls the interaction manner between the adsorbent and adsorbate. Also, the Temkin isotherm explains the adsorption behavior of heterogeneous systems. For adsorbents with homogenous surfaces, the Langmuir isothermal adsorption model is a standard monomolecular layer adsorption model that also shows that chemisorption dominates interaction between the adsorbent and adsorbate. The following equations represent the isotherm adsorption models [48,49].

(3) $$Freundlich\;model\;\;\log q_{e}=\frac{1}{n}\log C_{e}+\log K_{F}$$

(4) $$Langmuir\;model\;\;\frac{1}{q_{e}}=\frac{1}{q_{m\;}K_{L}}\;\frac{1}{C_{e}}+\frac{1}{q_{m}}$$

(5) $$Temkin\;model\;\;q_{e}=B\ln A_{T}+B\ln C_{e}$$

where q_e_ is the adsorption capacity (mg/g); q_m_ is the maximum adsorption efficacy (mg/g); K_F_ is the Freundlich constant (mg/g); K_L_ is the Langmuir constant (L/mg); C_e_ is metal ion equilibrium concentrations in medium (mg/L); n is the Freundlich exponent related to adsorption power (heterogeneity factor); B is constant heat of sorption; and A_T_ is the Temkin isotherm equilibrium binding constant (mg/mL).

**Figure 10:**
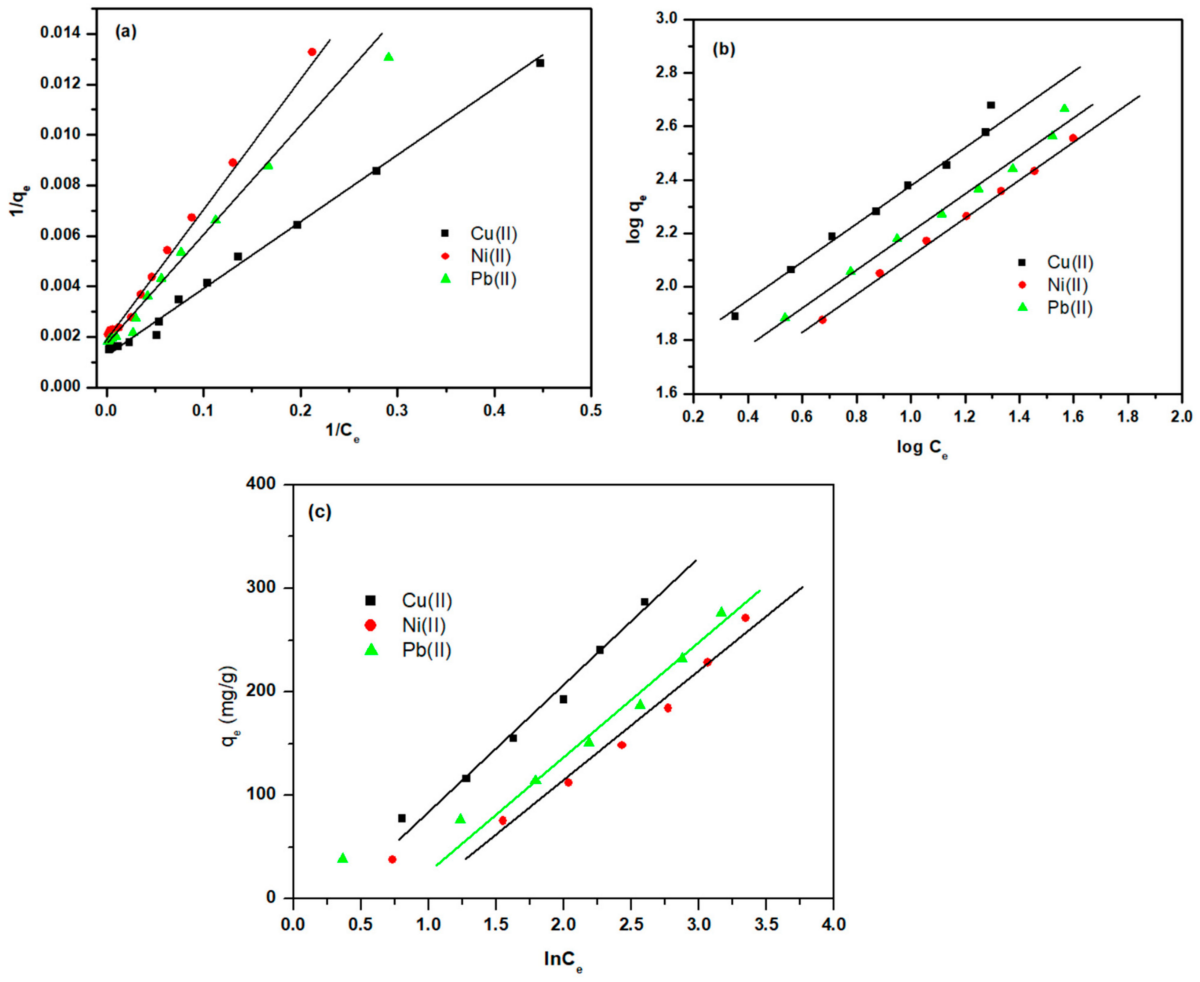
Langmuir (**a**), Freundlich (**b**), and Temkin (**c**) isothermal models for the adsorption of Cu(II), Ni(II), and Pb(II).

Figure 10a–c and Table 1 display the fitting curves and parameters for the isothermal models, respectively. Based on correlation coefficient values (R^2^), the Langmuir adsorption isotherm was determined to be the most suitable model since its R^2^ values are greater than those of the Freundlich and Temkin models. This suggests that a monolayer of metal ions chemisorbs onto the surface of the grafted copolymer. Furthermore, according to the Langmuir model, the quantities of Cu(II), Ni(II), and Pb(II) ions are adsorbed on the Cell-Jef148 surface’s adsorption active sites.

According to the Langmuir model fitting, the maximal adsorption capacities of Cu(II), Ni(II), and Pb(II) using Cell-Jef148 are 952.38 mg/g, 609.76 mg/g, and 769.23 mg/g, respectively. In the relevant results fitted by the Freundlich model, the value of n, the characteristic constant for the adsorption of Cu(II), Ni(II), and Pb(II) by Cell-Jef148, was 1.301, 1.395, and 1.403, respectively, and all of them were higher than 1, suggesting that the adsorption process is not complicated and belongs to preferred adsorption. A higher value of B in the Temkin isotherm adsorption model implies a more substantial variation in heat adsorption with adsorbent saturation and a quicker drop in adsorption heat during the adsorption process. Additionally, the fast variation in the heat adsorption was recorded with the adsorption of Cu(II).

#### 3.3.6. Adsorption Kinetic Studies

**Figure 11:**
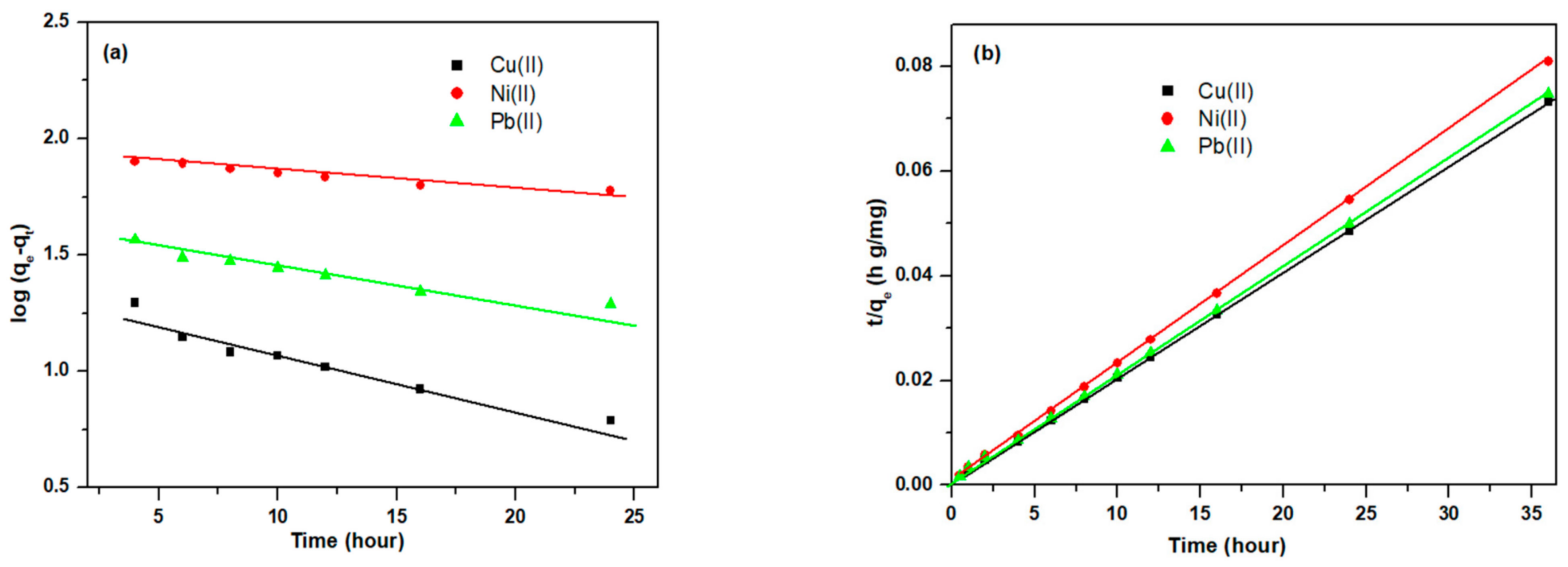
Linear plots of (**a**) pseudo-first-order and (**b**) pseudo-second-order models for adsorption of Cu(II), Ni(II), and Pb(II) metal ions onto Cell-Jef148.

The adsorption kinetics of Cell-Jef148 towards CU(II), Ni(II), Pb(II) metal ions were examined using kinetics models of pseudo-first-order and pseudo-second-order. The different kinetic parameters listed for Cell-Jef14 m (1 gm/L) are shown in Table 2. Figure 11a,b represents the linear plot of pseudo-first-order and pseudo-second-order models using the following Equations (6) and (7), respectively [50,51].

(6) $$Pseudo-First\;order\;Model\;\;\log\left(q_{e}-q_{t}\right)=\log q_{e}-\frac{K_{1}}{2.303}\;t\;\;\;\;\;\;\;$$

(7) $$Pseudo-Second\;order\;model\;\;\frac{t}{q_{t}}=\frac{1}{K_{s}\;\;q_{e}^{2\;}}+\frac{1}{q_{e}}\;t\;\;\;\;\;\;\;\;\;\;\;\;\;\;\;\;\;\;\;$$

where q_e_ and q_t_ are the adsorption capacities at equilibrium and at different time t (hour), respectively; K_1_ is the rate constant of pseudo-first-order; and K_s_ is the rate constant of pseudo-second-order.

As shown in Table 2, the R^2^ values of the pseudo-first-order model were 0.96709, 0.97199, and 0.96980 for Cu(II), Ni(II), and Pb(II), respectively. In contrast, the R^2^ values of pseudo-second-order were 0.99994, 0.99996, and 0.99990 for Cu(II), Ni(II), and Pb(II), respectively. Compared to the pseudo-first-order, the pseudo-second-order fitted the best data, confirming that the monolayer chemisorption governed the adsorption, which is well agreed with the Langmuir isothermal model. Moreover, the values of calculated q_e_ from pseudo-second-order are 497.5, 450.4, and 487.8 mg/g for Cu(II), Ni(II), and Pb(II), respectively, which are very close to the experimental q_e_ values. The outcomes from these results showed that the mechanism of pseudo-second-order adsorption is prevalent for Cu(II), Ni(II), and Pb(II) metal ion adsorption onto the Cell-Jef148 sorbent. All of the results obtained from the iosotherm adsorption models and pseudo-second-order models suggest that the addition of -NH_2_ groups to the cellulose surface may be responsible for the higher metal ion adsorption capacity of Cell-Jef148.

#### 3.3.7. Mechanism of Heavy Metal Ion Adsorption

**Figure 12:**
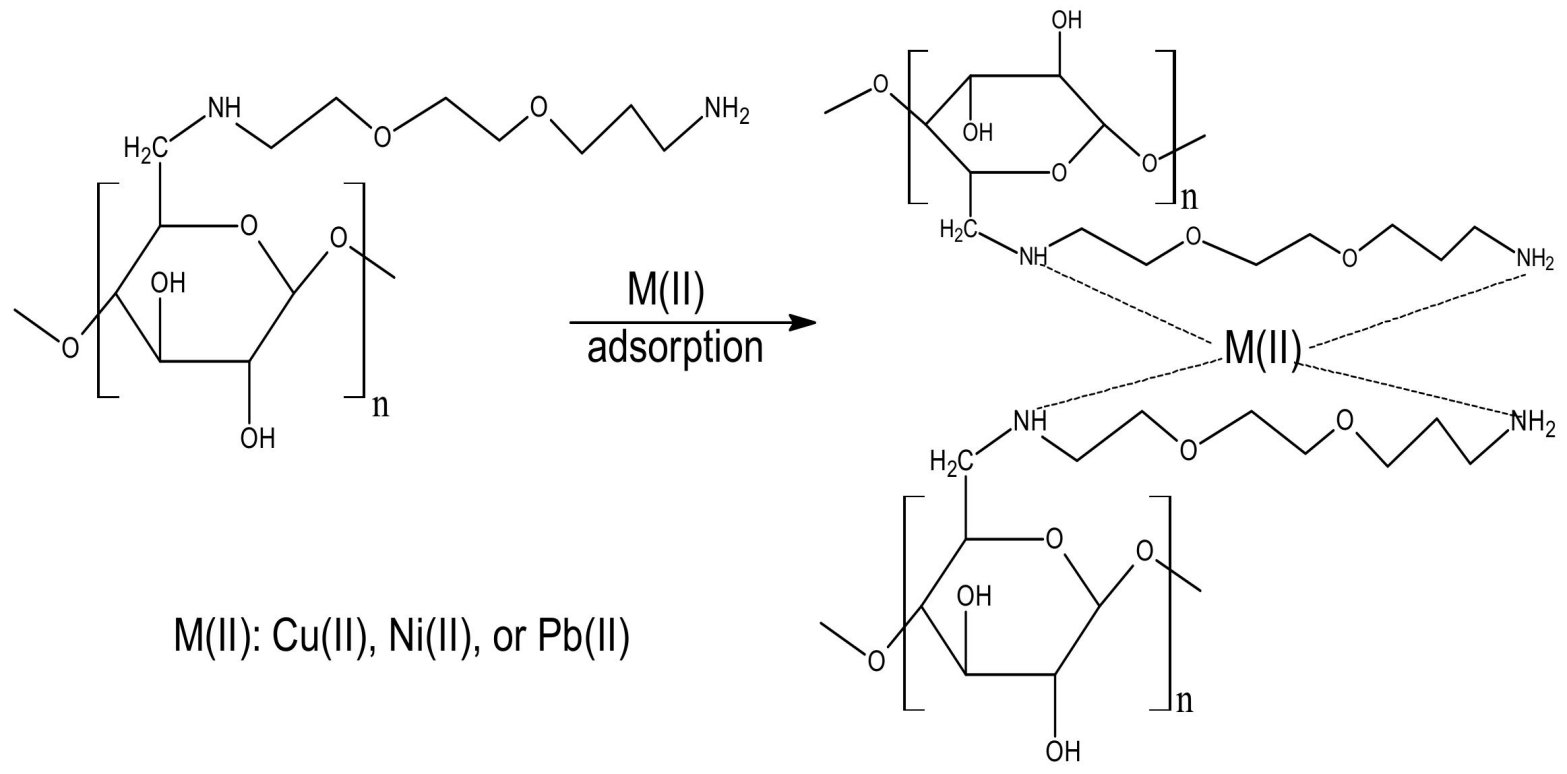
The proposal mechanism of adsorption of heavy metal ions onto Cell-Jef148.

Heavy metal ion adsorption onto Cell-Jef14 adsorbent can arise by several types of interactions at the adsorbent interface, such as ion exchange, electrostatic interactions or coordination of the metal ion by the functional groups (–OH, –NH, and –NH2). In our study, FTIR (Figure 2B) provides a suitable explanation for the adsorption of Cu(II), Ni(II), and Pb(II) ions onto Cell-Jef148. As shown in FT-IR spectra (Figure 2B), the stretching vibrations of -OH and -NH_2_ bands slightly changed with decreasing intensity. Moreover, a slight shift of peak at C-N group was recorded after adsorption of the metal ions. These results suggested that there is a strong electrostatic interaction between Cell-Jef48 and the heavy metal ions [52,53], not coordination linkage, as shown in the following Figure 12.

#### 3.3.8. Desorption and Regeneration of Cell-Jef148 Adsorbent

**Figure 13:**
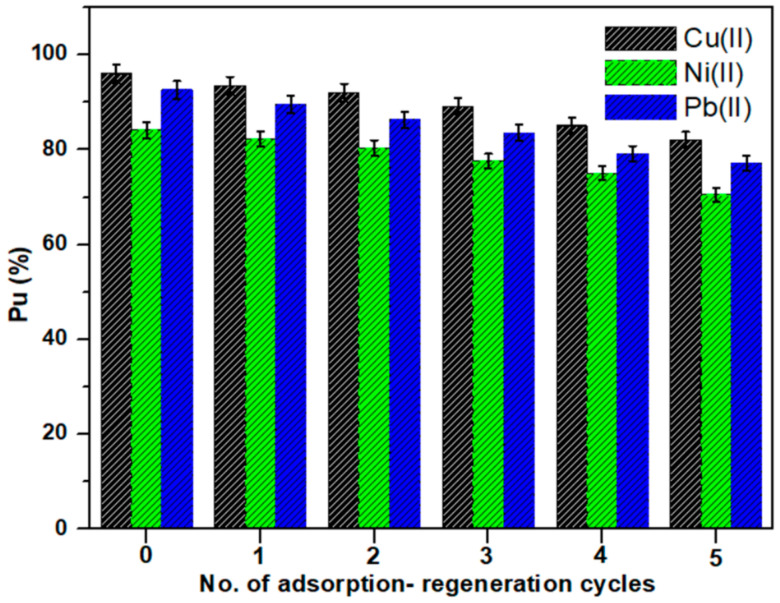
The percent uptake of Cell-Jef148 after several regeneration cycles.

The evaluation of the desorption efficiency and reusability of Cell-Jef148 is necessary for industrial applications. The regeneration of Cell-Jef148 was studied for repeated adsorption of the heavy metal ions during six cycles using 0.2 M HNO_3_ as desorption reagent [26]. Figure 13 showed the adsorption ratio of regeneration adsorption by Cell-Jef148 for five cycles. As observed in Figure 13, the adsorption capacities of Cell-Jef148 for Cu(II), Ni(II), and Pb(II) were 82.1%, 70.5%, and 77.2% at the fifth cycle of regeneration, while the adsorption capacities at the first cycle of regeneration were 93.5%, 82.3%, and 89.6%, respectively. Accordingly, Cell-Jef148 lost from its initial adsorption capacity about 13.96%, 13.6%, and 15.44% for Cu(II), Ni(II), and Pb(II), respectively, after five cycles of desorption. All these results indicate that Cell-Jef148 has high stability with a high ability for regeneration and reuse as an adsorbent for removing heavy metal ions. Therefore, Cell-Jef148 could be employed as an affordable and eco-friendly adsorbent to eliminate the heavy metal ions from wastewater.

#### 3.3.9. Comparative Studies of Cell-Jef148 with Previous Studies

Table 3 lists the Cell-Jef148 sorbents’ relative adsorption capacities for the removal of Cu(II), Ni(II), and Pb(II) in comparison to other reported similar sorbents. The data listed in Table 3 show that the adsorption efficiency of Cell-Jef148 examined in this work is preferable compared to those found in the referenced studies. These results suggest that this method of converting cellulose to Cell-Jef148 can result in a powerful adsorbent for the elimination of heavy metals, which is consistent with the sustainability concepts.

## 4. Conclusions

In this work, cellulose was dissolved in BMIMCl ionic liquid to prepare cellulose with an active Cl group (Cell-Cl) to be able to react with jeffamine EDR148 and produce new adsorbent material for removing heavy metal ions from the aqueous solutions. The structure of Cell-Jef148 was characterized using different techniques such as SEM, FT-IR, solid-state ^13^C-NMR, X-ray, and TGA to confirm the successful modification of cellulose with jeffamine EDR148. To remove Cu(II), Ni(II), and Pb(II) from aqueous media, the adsorption behaviors of Cell-Jef148 were examined, and the most efficient adsorption conditions were evaluated to be as follows: pH 5.5, an adsorption duration of 4 h, a 500 mg/L metal ion concentration, a 1 gm/L adsorbent dose, and a temperature of 30 °C. The adsorption behaviors of Cell-Jef148 were fitted with the Langmuir isothermal model and the pseudo-second-order model, which describe how metal ions interact chemically with Cell-Jef148. In five successful adsorption/desorption cycles, it was proved that the Cell-Jef148 adsorbent can be effectively reused with higher adsorption effectiveness of Cu(II), Ni(II), and Pb(II) ions. Based on the observed outcomes, it was determined that newly aminated cellulose with jeffamine EDR148 is a very promising bio-sorbent material for heavy metal removal. As a result, the eco-friendly Cell-Jef148 can be used to remove organic and inorganic pollutants in the wastewater treatment with high adsorption capacity, affordable cost, and recyclable sorbent.

## Figures and Tables

**Table 1:** Adsorption isotherm parameters for the heavy metal ion adsorption onto Cell-Jef148.

	Langmuir Isotherm			Freundlich Isotherm			Temkin Isotherm		
	Constants			Constants			Constants		
	K_L_ (L/mg)	q_m_ (mg/g)	R^2^	K_F_ (L/mg)	n	R^2^	K_T_Mg/L	B	R^2^
Cu(II)	0.0406	952.38	0.99795	4.953	1.301	0.97949	0.6597	136.572	0.96604
Ni(II)	0.0307	609.76	0.99855	4.096	1.395	0.98011	0.4719	104.245	0.95662
Pb(II)	0.0323	769.23	0.99575	4.437	1.403	0.97785	0.6772	99.017	0.95364

**Table 2:** Adsorption kinetic constants of pseudo-first-order and pseudo-second-order models for heavy metal ion adsorption onto Cell-Jef148.

	Pseudo-First-Order			Pseudo-Second-Order			
	Constants			Constants			
	K_1_ (L/mg)	q_e_ (mg/g)	R^2^	K_s_ (L/mg)	q_e_ (mg/g)	R^2^	q_e_ (Exp.) (mg/g)
Cu(II)	0.05262	20.2889	0.96709	8.365 × 10^−3^	497.513	0.99994	480.3
Ni(II)	0.01519	83.5795	0.97199	4.785 × 10^−3^	450.451	0.99996	420.4
Pb(II)	0.03047	38.2798	0.96980	5.163 × 10^−3^	487.805	0.99990	463.2

**Table 3:** Adsorption capacity comparison of the removal of Ni(II) and Cu(II) for various adsorbents.

The Adsorbent	Heavy Metal Ion M(II)	Initial Metal Ion Concentration (C_i_)	q_max_, (mg/g)	References
Cellulose o-pheneylendiamine polymer	Pb(II)	600	406.8	[54]
Cyclodextrin/carboxymethyl cellulose composite	Pb(II) Cu(II)	500 200	393.56 158.12	[49]
Grafted polyethylenimine (PEI) onto carboxylated microcrystalline cellulose (SA-MCCMV),	Pb(II)	500	357.1	[55]
Hydroxyethyl cellulose (HEC)-grafted EDTA	Pb(II) Cu(II)	400 400	190 100	[56]
Cellulose–jeffamine EDR148	Pb(II) Ni(II) CU(II) CU(II)	500 500 500 200	480.3 420.5 471.9 192.6	Current work

## Data Availability

All data analyzed during this study are included in this published article.

## Supplementary Materials

The following supporting information can be downloaded at: https://www.mdpi.com/article/10.3390/polym17020255/s1. Figure S1. Influence of pH on the adsorption of the Heavy metal ions onto (a) Pure-cell and (b) Cell-Cl at adsorption conditions of 500 mg/L metal ion concentration, 50 mL volume of the adsorption solution, 50 mg of the adsorbent, temperature of 25 °C, and time of 4 h; Figure S2. Influence of temperature on the adsorption of the Heavy metal ions onto (a) Pure-cell and (b) Cell-Cl at adsorption conditions of 500 mg/L metal ion concentration, 50 mL volume of the adsorption solution, 50 mg of the adsorbent, pH of 5.5, and time of 4 h. Figure S3. Influence of time on the adsorption of the Heavy metal ions onto (a) Pure-Cell and (b) Cell-Cl at adsorption conditions of 500 mg/L metal ion concentration, 50 mL volume of the adsorption solution, 50 mg of the adsorbent, temperature of 25 °C, and pH of 5.5. Figure S4. Influence of metal ion concentration on the adsorption of the Heavy metal ions onto (a) Pure-cell and (b) Cell-Cl at adsorption conditions of 50 mL volume of the adsorption solution, 50 mg of the adsorbent, temperature of 25 °C, pH of 5.5, and time of 4 h.

## Abbreviations

The following abbreviations are used in this manuscript:

BMIMCl	N,N-butylmethylimidazolium chloride
SEM	Scanning electron microscopy
FT-IR	Fourier transform infrared
solid-state ^13^C-NMR	Solid-state ^13^C-nuclear magnetic resonance spectroscopy (CP/MAS ^13^C-NMR)
XRD	X-ray diffraction
TGA	Thermogravimetric analysis
Pure-Cell	Cellulose cotton
Cell-Cl	6-Chlorodeoxycellulose
Cell-Jef148	Aminated cellulose with jeffamine EDR148
q_e_	Adsorption capacity
Pu	Percent uptake
