# Supplementary material for: Characterization and Adsorption Behavior of Newly Synthesized Aminated Cellulose with Jeffamine EDR148 Towards Ni(II), Cu(II), and Pb(II) Heavy Metal Ions
Source: Polymers (Basel). 2025 Jan 20;17(2):255. doi: 10.3390/polym17020255 (PMC11768622; doi:10.3390/polym17020255)
Supplement: Supplementary file 1 [file polymers-17-00255-s001.zip › polymers-3411759-supplementary.pdf]

Characterization and Adsorption Behavior of Newly Synthesized Aminated Cellulose with Jeffamine EDR148 towards Ni(II), CU(II), and Pb(II) Heavy Metal Ions

1. Characterization

1.1. Scanning electron microscopy (SEM):

With an accelerating voltage of 10 KV, the surface morphology of cellulose and the modified samples was analyzed using a Hitachi S-4100 SEM (Hitachi High-Tech, Tokyo, Japan).

1.2. Solid state NMR measurement (13C CP MAC NMR):

On a Delta2-NMR spectrometer (B0 = 9.4T) (Jeol Ltd. Tokyo, Japan) at Kyushu University, solid state 13C NMR investigation was performed with a resonance frequency of 100.52 MHz. The Aurum tube-capped zirconia rotors (6 mm) were used to hold the samples. Both the 5-KHz spinning speed and the decoupling with TPPM at γB1/2P = 100 kHz were used. Be aware that compatibility between the results obtained with both separate devices and settings was demonstrated by comparing the data for untreated cellulose.

1.3. Fourier Transform Infrared Spectroscopy (FT-IR spectra):

IR spectra of cellulose and its modified samples were recorded with 32 scans at a maximum resolution of 2 cm_{-1} using the Bio-Rad FTS 6000 spectrometer (Tokyo, Japan). Weighing 1.0 mg of sample pressed onto potassium bromide resulted in a layer of material that was 10 μm thick. The FT-IR spectrum's scanned range was 400–4000 cm^{-1}.

1.4. Thermogravimetric analysis (TGA):

Using the Seiko TG/DTA6300 (Seiko Instruments Inc., Chiba, Japan) in a nitrogen atmosphere, TGA was used to describe cellulose and modified samples with jeffamine EDR148. The samples measured weighed between 1.1 and 1.4 mg, and the temperature range for TGA was 28 to 530 °C, with a heating rate of 10 °C/min.

1.5. X-ray Measurements:

By using Rigaku RINT2100H/KLC X-ray diffraction equipment (Rigaku, Tokyo, Japan) with Ni-filtered Cu Kα radiation at ambient temperature and a 2θ range of 5° to 55°, the cellulose samples' X-ray diffractograms (XRDs) were recorded.

2. Results and Discussion

2.1. Adsorption behavior of the modified cellulose towards heavy metals

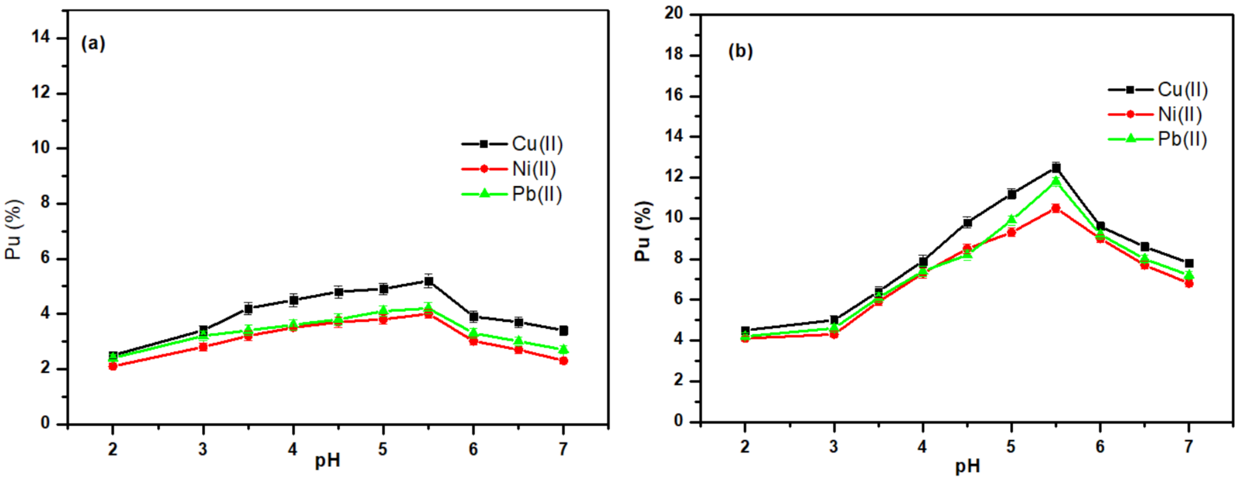

Figure S1. Influence of pH on the adsorption of the Heavy metal ions onto (a)Pure-cell and (b) Cell-Cl at adsorption conditions of 500 mg/L metal ion concentration, 50 mL volume of the adsorption solution, 50 mg of the adsorbent, temperature of 25 ◦C, and time of 4 hours.

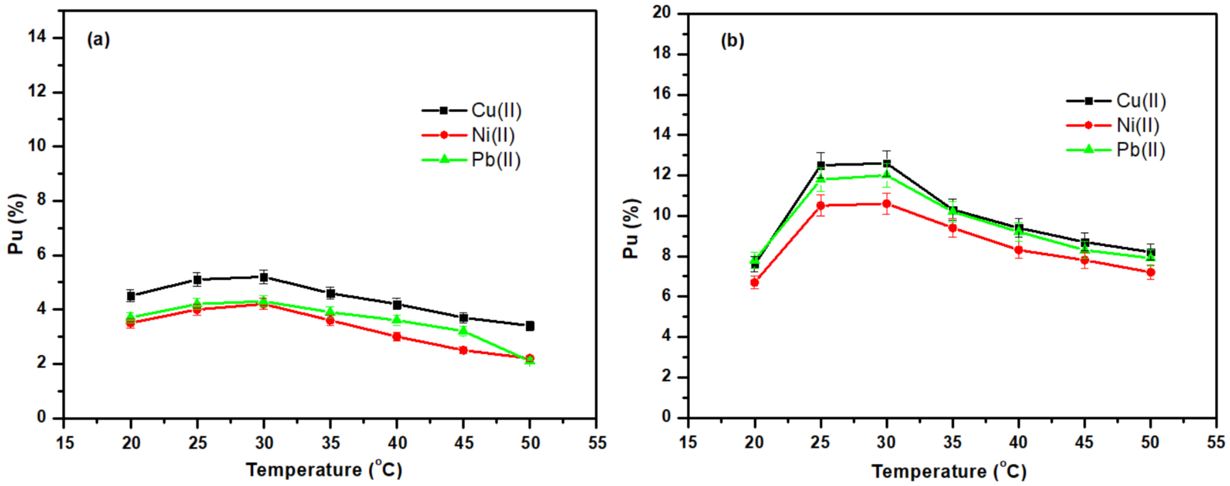

Figure S2. Influence of temperature on the adsorption of the Heavy metal ions onto (a) Pure-cell and (b) Cell-Cl at adsorption conditions of 500 mg/L metal ion concentration, 50 mL volume of the adsorption solution, 50 mg of the adsorbent, pH of 5.5, and time of 4 hours.

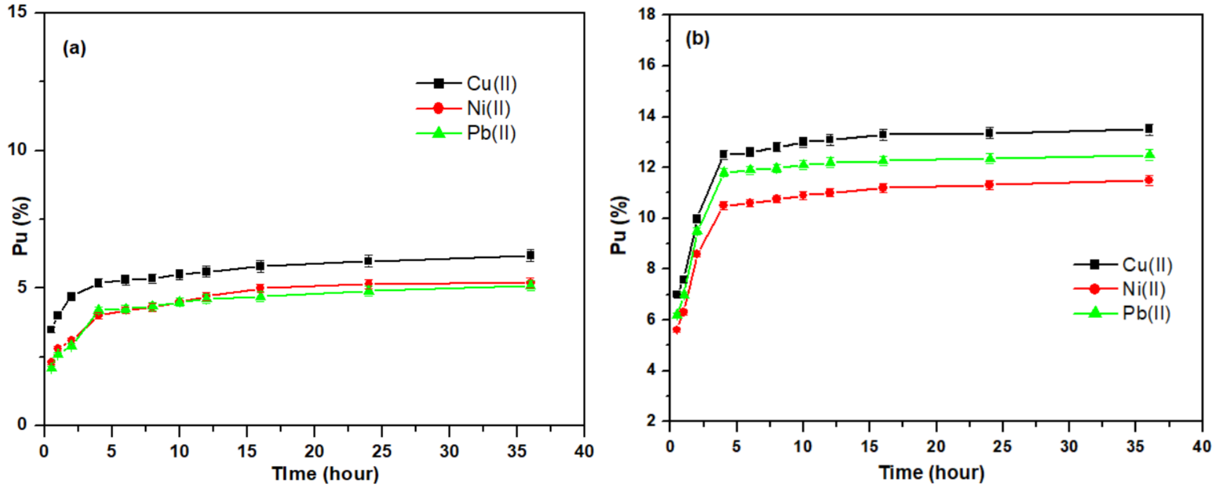

Figure S3. Influence of time on the adsorption of the Heavy metal ions onto (a) Pure-Cell and (b) Cell-Cl at adsorption conditions of 500 mg/L metal ion concentration, 50 mL volume of the adsorption solution, 50 mg of the adsorbent, temperature of 25 ◦C, and pH of 5.5.

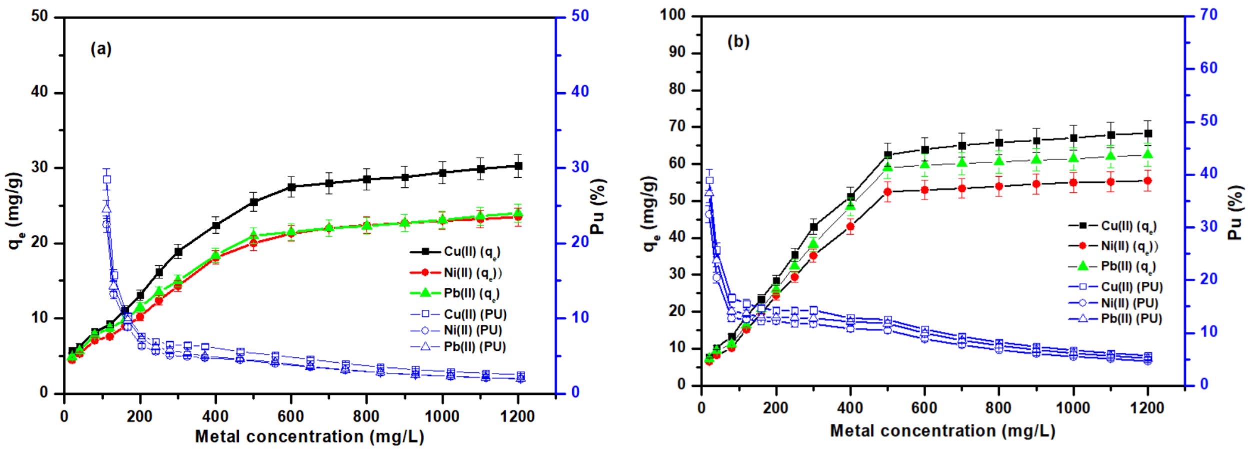

Figure S4. Influence of metal ion concentration on the adsorption of the Heavy metal ions onto (a) Pure-cell and (b) Cell-Cl at adsorption conditions of 50 mL volume of the adsorption solution, 50 mg of the adsorbent, temperature of 25 ∘C, pH of 5.5, and time of 4 hours.
